# Defining hospital community benefit activities using Delphi technique: A comparison between China and the United States

**DOI:** 10.1371/journal.pone.0225243

**Published:** 2019-11-20

**Authors:** Aijun Xu, Hossein Zare, Xue Dai, Yuanxi Xiang, Darrell J. Gaskin

**Affiliations:** 1 School of Nursing, Nanjing University of Chinese Medicine, Nanjing, Jiangsu, China; 2 Department of Health Policy and Management, Johns Hopkins Bloomberg School of Public Health, Baltimore, Maryland, United States; 3 University of Maryland University College, Health Services Management, Adelphi, Maryland, United States; 4 School of Management, Hubei University of Chinese Medicine, Wuhan, Hubei, China; Iran University of Medical Sciences, ISLAMIC REPUBLIC OF IRAN

## Abstract

**Introduction:**

Currently there is no expert consensus regarding what activities and programs constitute hospital community benefits. In China, the hospital community benefit movement started gaining attention after the recent health care system reform in 2009. In the United States, the Internal Revenue Service and the nonprofit hospital sector have struggled to define community benefit for many years. More recently, under the Affordable Care Act (ACA)’s new “community benefit” requirements, nonprofit hospitals further developed these benefits to qualify for 501(c)(3) tax exempt status.

**Methods:**

The Delphi survey method was used to explore activities and/or programs that are considered to be hospital community benefits in China and the United States. Twenty Chinese and 19 American of academics, senior hospital managers and policy makers were recruited as experts and participated in two rounds of surveys. The survey questionnaire was first developed in China using the 5-point Likert scale to rate the support for certain hospital community benefits activities; it was then translated into English. The questionnaires were modified after the first round of Delphi. After two rounds of surveys, only responses with a minimum of 70 percent support rate were accepted by the research team.

**Results:**

Delphi survey results show that experts from China and the U.S. agree on 68.75 percent of HCB activities and/ or programs, including *emergency preparedness*, *social benefit activities*, b*ad debt /Medicaid shortfall*, *disaster relief*, *environmental protection*, *health promotion and education*, *education and research*, *charity care*, *medical services with positive externality*, *provision of low profit services*, and *sliding scale fees*.

**Conclusions:**

In China, experts believe that healthcare is a “human right” and that the government has the main responsibility of ensuring affordable access to healthcare for its citizens. Meanwhile, healthcare is considered a commodity in the U.S., and many Americans, especially those who are vulnerable and low-income, are not able to afford and access needed healthcare services. Though the U.S. government recognized the importance of community benefit and included a section in the ACA that outlines new community benefit requirements for nonprofit hospitals, there is a need to issue specific policies regarding the amounts and types of community benefits non-profit hospitals should provide to receive tax exemption status.

## Introduction

Previous studies have examined hospital community benefits (HCB) and the role of hospitals in society [1–5]. However, a broader consensus has not been reached among academics and hospital administrators regarding which activities should be included in the definition of HCB. Considering the dynamic context of different countries and their health systems, national-level research is needed to fill the knowledge gap regarding HCB. This study aims to identify, examine, and compare specific HCB activities in China and the U.S. using the Delphi method. China and the U.S. have unique health systems, healthcare resource allocations, and HCB contexts. Findings from this research will provide a better understanding of the nature of hospital community benefits and a frame of reference for identifying HCB activities and/or programs at national or state level for countries all over the world.

Our study showed that there are several important similarities between US and China’s healthcare system, and there are lessons learned from both sides. For example, both countries are looking to reform their healthcare systems, address challenges associated with inadequate insurance coverage [6], and high healthcare costs for patients (e.g. coinsurance, deductible and other out-of-pocket expenditures) [7]. Despite these similarities, there are some significant differences between China and the U.S.: nonprofit hospitals covered 70 percent of hospital beds in the U.S. compared to less than 20 percent in China [7]. Considering the two countries’ similar but different healthcare contexts, the current study aims to examine commonalities and discrepancies of HCB activities in China and the U.S, from perspectives of academic experts, hospital administrators, and policy makers.

China’s health system had a transition from a centrally planned to a market-oriented one in less than twenty years, and its health outcome and system performance also gained unprecedented progress [8]. Even though life expectancy has risen and infant mortality has plummeted significantly over the last 20 years [9], concerns had been raised on how to reform its health care delivery and payment system; health care resources are unequally distributed across the country—urban and rural disparities in health care quality and access still exist [8]. The country also lacks an effective primary care system and the cost of health care is often too high [9].

In 2009, China launched a nationwide healthcare system reform with a goal to provide affordable, equitable access to essential healthcare services for all its citizens by 2020 [8, 10]. From the payment system perspective, the Chinese government implemented policies to provide universal health insurance coverage to all of its citizens. As a result, 95 percent of the Chinese population– 1.3 billion people–were covered by one of the public social insurance programs at the end of 2011 [11]. From the delivery system perspective, China went through an attempted transformation, moving from a fragmented, profit-driven public/private hospital-centered system to an integrated primary care-based delivery system [12, 13].

Although the healthcare payment and delivery system reforms made considerable progress, a lot of work remains to be done to provide accessible and affordable to citizens across the country. Public hospitals are still partially profit-driven, so they shifts focus from prescription drugs to clinical examination and diagnostic tests [14]. Patients who have limited medical coverage cannot afford skyrocketing medical and prescription drug costs. Because of these urgent issues, hospital assessment policies released by the Chinese Ministry of Health in 2011 and 2012, listed fulfilling public interest as the primary function of public hospitals [15]. Public hospitals, which comprise the majority of all hospitals in China [10], are expected to respond quickly and are accountable for the government’s mandates to environment protection and establish more facilities and services for emergencies.

HCB is becoming increasingly important both inside and outside of the health industry in China. However, the concept of HCB is still in its initial development stage in China and the scope and activities of hospital community benefits have not been standardized at the national level.

Healthcare services in the U.S. are delivered through various types of providers and hospitals and financed through third party public (e.g. Medicare and Medicaid) and private payers. In 2015 approximately 58.5 percent of the 4,862 community hospitals were non-profit, 20.2 percent were public and 21.3 percent were investor-owned [16]. Non-profit hospitals are exempt from most federal, state and local taxes, in acknowledgement of the “community benefits” they provide [3, 17].

Previously, section 501(c)(3) of the Internal Revenue Code required non-profit hospitals to meet the “community benefits” standards to qualify for tax-exempt status [18]. Spending on charity care and activities that promote community health are considered meeting the requirements for tax exemption. The Internal Revenue Services (IRS), however, allows hospitals to determine broadly what activities and services count towards community benefits [19]. This resulted in significant variation in the definition of a community benefit activities and/or programs and how their value was measured.

Public debate over whether non-profit hospitals provided adequate community benefit activities in exchange for their tax-exempt status culminated in the enactment of new community benefit requirements under the Affordable Care Act (ACA). In 2008, the IRS had revised Form 990 and Schedule H for the first time since 1979. The agency added new requirements for hospitals to submit additional information regarding community benefits on the new schedule H worksheet attached to form 990, which must be filed annually with the IRS in order to maintain their tax exempt status [20]. These new requirements were designed to improve transparency and accountability, and to support an overall strategy to improve preventative care and population health through community health initiatives outlined in the ACA.

As of 2009, the IRS began requiring non-profit hospitals to document their community benefits in a consistent manner. The IRS defines community benefits as the sum of charity care; unreimbursed costs from Medicaid and other public means-tested programs; community health promotion and health education services; subsidized medical services, research, community advocacy, and community building activities; health professional education; and cash and in-kind contributions to community-based organizations [21]. In addition to these national community benefits standards established by the IRS, hospitals must consider state laws on community benefits with substantially varied definitions, populations served, and quantitative methods used to determine the costs [20, 22, 23].

### Definition and identification model of hospital community benefits

Researchers and healthcare organizations in the both countries have attempted to define the scope and activities of hospital community benefit–as those scholars pointed out, HCB includes activities beyond uncompensated care [18, 24–32]. Table 1 provides more details on HCB activities.

**Table 1:** HCB activities or programs defined by researchers or organizations.

Authors/Organization	Activities Defined as Community Benefits
**China**	
**Weijun** [33], **Lei** [32]	Charity Care Uncompensated preventative services (i.e. annual check-ups) Village visits Uncompensated medication delivery Cost of bad debts
**Chinese Academy of Social Sciences** [26]	Fair distribution of medical services Public health services Trusting patient-provider relationships Avoidance of conflict of interest with the industry Environmental health Research and education
**Shanghai Spiritual Civilization Office** [28]	Employer responsibility Service accountability Fiduciary responsibility Community service Environmental health
**United States**	
**IRS** [18]	Charity Care Unreimbursed Medicaid Services (Medicare Shortfalls) Unreimbursed Other (e.g. costs of other means-tested government programs) Community Benefit Services (e.g. community health improvement services and community benefit operations) Unreimbursed Education (e.g. health professions education) Health Services (Not means-tested, e.g. subsidized health services) Unfunded Research Activities Community Benefit Contributions (e.g. cash and in-kind contributions for community benefit)
**Centers for Medicare & Medicaid Services** [29]	Medicare includes all IRS items except unreimbursed education. It also includes bad debt.
**Catholic Health Association** [31]	Community health improvement services Health professions education activities or programs Subsidized health services Research programs Cash and in-kind contributions Community-building activities Community benefit operations
**American Hospital Association (AHA)** [30]	AHA includes all activities in the CHA definition. It also considers bad debt expense and unreimbursed Medicare costs as part of community benefit
**Zimmerman** [24]	Cost of services provided without charge to persons with no or a limited ability to pay Cost of bad debts Cost of Medicaid services in excess of Medicaid reimbursement cost of services to improve community members’ health who are medically underserved and disadvantaged
**Nicholson** [25]	Uncompensated care Cost of other charitable public-good services Losses on medical research, taxes, Medicaid and Medicare shortfalls Price discounts to privately insured patients, and losses on medical education
**Ginn** [27]	Uncompensated care Services that have benefits beyond their direct recipients Research and education Unrestrictive access to services Community health

In the U.S., the Catholic Health Association (CHA) has been the leading expert in the community benefit field. The IRS heavily adopted CHA’s recommendation in developing its reporting requirements for all public hospital community benefit activities. The American Hospital Association (AHA) also relied on CHA’s definition to develop its guidance on community benefit reporting guidelines [34]. Thus, for the purpose of this study, CHA’s definition is used to describe community benefits as activities or programs that provide care and/or promote community health in response to identified community needs [31].

**Fig 1:**
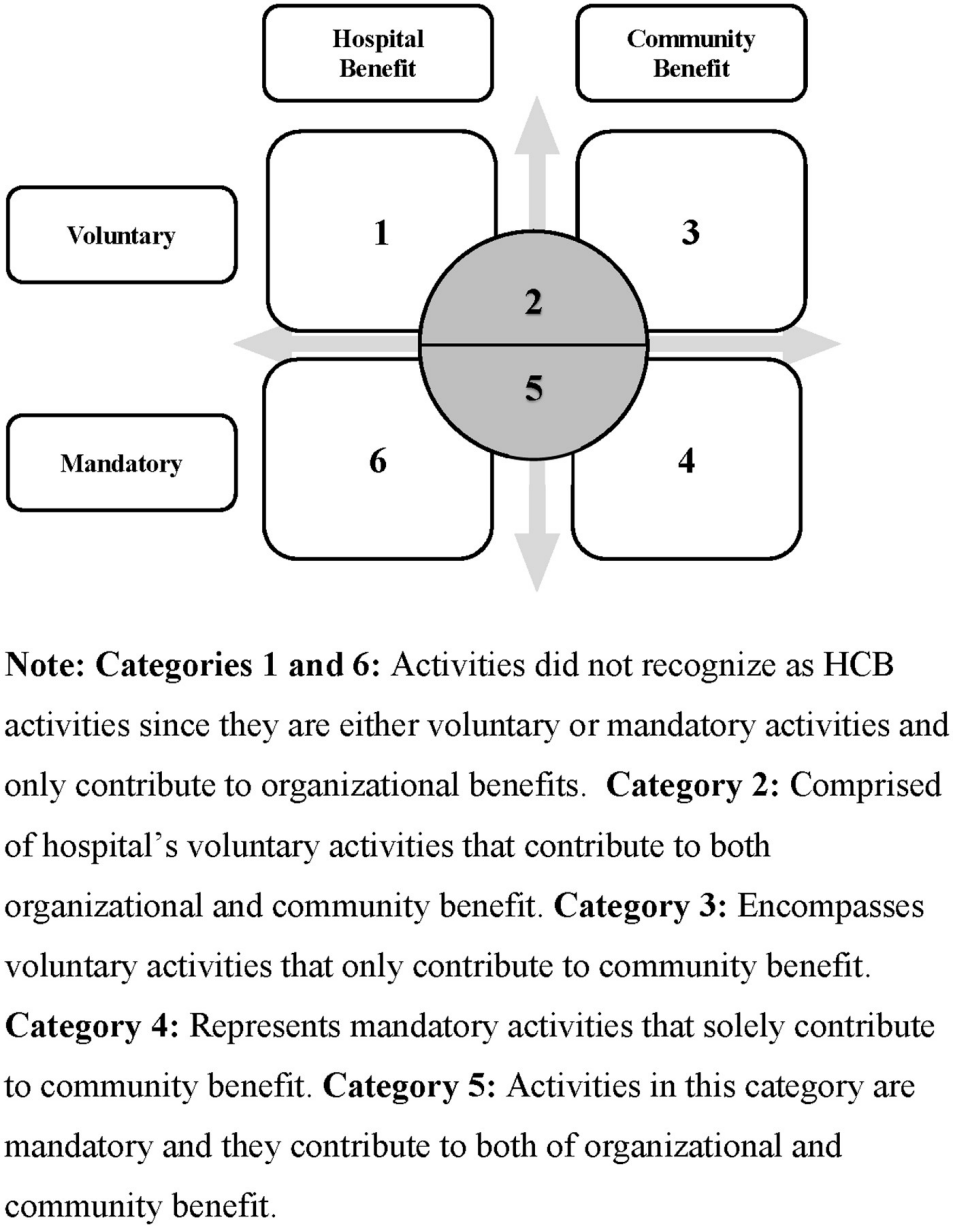
Hospital community benefit classification model.

Given HCB’s wide scope, a Hospital Community Benefits Identification Model (Fig 1) was developed to identify and classify HCB activities [35]. The model divides these activities/programs into six categories with two dimensions–their nature and direct outcome. They characterize the first dimension as either mandatory or voluntary. The second dimension is classified as hospital benefits and/or community benefits. Hence, all hospital activities and programs can be classified into six categories and out of these categories, four of them—2, 3, 4 and 5—are considered HCB activities.

Based on the above conceptual framework, the questionnaire was developed to compare expert opinions of HCB activities in China and the U.S. using the Delphi method.

## Materials and methods

The Delphi method is a qualitative technique that allows experts, who come from a variety of background to formulate a list of ideas and then come to a consensus regarding their relative importance [36, 37]. For this study, an expert was defined as someone who is knowledgeable about the subject matter and is capable of representing the views of his or her peers. Experts who participated in the study responded anonymously to two rounds of structured questionnaires. Double-blinded correspondence interviews were conducted to ensure minimizing the peer pressure and encouraging free exchange of ideas [38]. The study was approved by the Research Ethics Committee of Nanjing University of Chinese Medicine and The Johns Hopkins Bloomberg School of Public Health Institutional Review Board.

### Questionnaire

A content analysis was conducted on the website news of 150 hospitals in China on the basis of literature review and HCB Identification Model. Eight HCB activities generated from literature, including “undertaking rescues under emergencies” and another eight HCB activities generated from website news coverage including “provide excellent medical services". After the integration of these two studies’ results, we construct HCB system including 14 kinds of activities in Chinese context [4].

On the questionnaire, experts were asked to rate the degree of support for listed HCB activities on a five-point scale (strongly disagree, disagree, neither agree nor disagree agree, and strongly agree). The first round of the survey included 14 Likert scaled questions for HCB activities. Supplemental materials about the purpose, scope, and framework of the study were also provided to experts. After analyzing the first round responses, the questionnaire was modified for the second round of surveying, which included 13 Likert scaled questions and one open question for experts’ suggestions (See Table 2).

**Table 2:** Time period and final participants of Delphi study.

Delphi rounds	Round Objective	Study in China	Study in the USA
		Time Period	Time Period
**Round 1**	Get the agreement rate for each activity/ program	20, Sept -10 Oct, 2010	10, July–2 Sep, 2014
**Round 2**	Revise the questionnaire of each activity/ program according to the agree rates. Hand out the modified questionnaire and get the agree rate once more.	8–22 Nov, 2010	19 Sep–11 Nov, 2014
**Final Participants**		**Participants in China(N = 20)**	**Participants in USA(N = 19)**
	Academic	10	10
	Senior Managers (Hospitals)	7	7
	Policy Maker	3	2

**Sources**: Study findings; China: 2010, USA: 2014; Note: In China 20 experts participated in first and second Delphi and in the U.S. 19 experts participated in first round and 13 experts responded second Delphi.

A Cronbach’s alpha was computed and the estimated correlation for the final survey were 0.819 in Chinese study and 0.9641 in the U.S., which demonstrate that the HCB activities have defined in the questionnaire, were well correlated.

### Delphi experts, data collection and Delphi process

Panel expert discussions for the Delphi research started from September 2010 in China, July 2014 in US, to February 2011 and December 2014, respectively. The expert panels included 20 experts in China and 19 experts in the U.S. who were policy makers, hospital senior managers, and academic experts. (See Table 2). Before the expert discussion, informed consent was obtained. Non-human subject studies do not require IRB approval based on the IRB’s policies of Nanjing University of Chinese Medicine and the Johns Hopkins University.

#### Data collection and Delphi process in China

For the first round, copies of the questionnaires were emailed and postage mailed to the experts who had been contacted and confirmed in advance. Twenty business days later, 100 percent of the questionnaires were returned. Results from the first round were disclosed to the experts and the second round of the Delphi study was carried out one month later to provide more time for experts to further elaborate their reasoning and revise their answers. Twenty copies of revised questionnaires were emailed and postage mailed to the experts. The response rate was 100 percent in China.

#### Data collection and Delphi process in the U.S.

During the first round, questionnaires with supplement documents were sent to 70 eligible experts (45 scholars based on their publication, 18 hospital HCB experts from Maryland Hospitals, and 8 Maryland policymakers) and 46 eligible experts agreed to participate in our study.

After three reminders with 15-day intervals between reminders, 19 experts responded (10 scholars, 7 hospital HCB experts, and 2 policy makers) to the first round. The revised questionnaires and the results of the first round were sent to these 19 experts. After three reminders, 13 experts completed the questionnaire; the response rate was 68.4 percent.

Qualtrics Data Collection Web-based Survey was used for data security purpose. Qualtrics is the most trusted enterprise research platform in the world with over 8,500 brands, it also uses Transport Layer Security (TLS) encryption (also known as HTTPS) for all transmitted data [39]. The survey was administered by the Hopkins Center for Health Disparities Solution with capacity to use by Mobile or Computer.

## Results

### Delphi first round

The literature suggests that in order to maintain the consistency of Delphi finding, only results with response rates of 70 percent should be accepted [40]. Other healthcare researchers have also used this benchmark rate [41, 42]. For the purpose of this study, a benchmark response rate of 70 percent was used.

Panel A included in Table 3 compares results of the first-round Delphi between China and the U.S. From 13 literature generated HCB activities, 7 had over 70 percent support rate and were supported by both Chinese and U.S. experts. These activities included: “*bad debt*,” “*emergency preparedness or disaster relief*,” “*provision of low profit services*,” “*charity care*,” “*education and research*,” “*health promotion and health education*,” “*social benefit activities*.*”* However, *“environmental protection”* and *“discounted pricing”* were supported by Chinese experts but not their U.S. counterparts.

**Table 3:** Support rates of the first Delphi round in China.

China			The United States			
*Panel A*: *First Round*: *Delphi*						
HCB Activities	Support rate	Mean (N = 20)	St. Dev.	Support rate	Mean (N = 19)	St. Dev.
1. High quality medical services	90%	4.63	0.83	58%	3.53	1.26
2. Bad debt	70%	3.68	0.89	74%	3.79	1.47
3. Emergency preparedness or disaster relief	100%	4.89	0.32	74%	4.05	0.91
4. Adhering/complying with government mandates	100%	4.63	0.50	32%	2.79	1.32
5. Environmental protection	95%	4.68	0.58	47%	3.47	1.02
6. Tax payments or payments in lieu of taxes	60%	3.68	1.00	53%	3.26	1.37
7. Medical services with positive externality	80%	4.37	0.83	68%	3.95	0.78
8. Discounted pricing	75%	3.89	0.94	42%	2.89	1.24
9. Provision of low profit services (e.g. trauma care)	75%	4.11	0.94	84%	4.11	0.99
10. Charity care	95%	4.63	0.50	100%	4.84	0.37
11. Education and research	95%	4.42	0.61	89%	4.37	0.84
12. Health promotion and health education	100%	4.63	0.50	95%	4.53	0.61
13. Social benefit activities	80%	4.21	0.85	95%	4.47	0.61
***Panel B*: *2***^***nd***^ ***Round Delphi***	**Support rate**	**(N = 20)**	**St. Dev**.	**Support rate**	**(N = 13)**	**St. Dev**.
1. High quality medical services	95%	4.65	0.60	21%	2.5	1.09
2. Bad debt ^(a)^	90%	4.10	0.50	64%	3.5	1.33
3. Emergency preparedness or disaster relief (USA: Emergency preparedness above what is required for license) ^(b)^	100%	4.85	0.37	93%	4.36	0.84
4. Adhering/complying with government mandates	100%	4.85	0.37	14%	2.43	1.28
5. Environmental protection (USA: Environmental protection that directly affect the health of population, above what is required for license)	100%	4.70	0.48	93%	4.0	0.78
6. Tax payments or payments in lieu of taxes	75%	3.75	1.09	36%	3.14	1.29
7. Medical services with positive externality	95%	4.45	0.61	71%	3.79	1.05
8. Discounted pricing (USA: Sliding scale based on income)	80%	4.10	0.97	86%	3.93	0.73
9. Provision of low profit services (e.g. trauma care)	90%	4.35	0.67	71%	4.0	0.96
10. Charity care	100%	4.65	0.50	100%	4.71	0.47
11. Education and research	100%	4.70	0.48	71%	4.07	1.00
12. Health promotion and health education	100%	4.75	0.42	79%	4.21	0.80
13. Social benefit activities	90%	4.35	0.68	93%	4.29	0.83
14. Medicare shortfall ^(c)^	n/a	n/a	n/a	29%	2.86	1.03
15. Medicaid shortfall ^(c)^	n/a	n/a	n/a	71%	3.86	0.86

**Source**: Study findings; China: 2010; Notes: For each question, it was a Likert scale moved from strongly disagree (1) and strongly agree (5), the support rate computed if experts responded 4 or 5.

^(a)^ For USA; there were 2 indicators for bad debt: 1) Bad debt to low-income group (Mean: 3.57, SD:1.50, SR:64%) and Bad debt because of catastrophic expenditure (Mean: 3.43, SD:1.28, SR: 57%), For this table we reported the average of two indicators.

^(b)^ For the U.S. we asked disaster relief above what is required for license as separate questions (Mean: 4.29, SD:0.83, SR: 79%),

^(c)^ non-applicable for China

### Delphi second round

For the second round of Delphi, after taking experts’ suggestions from the first round, the U.S. questionnaire was modified. For example, “*bad debt*” was divided into “*bad debt for low-income groups*” and “*bad debt because of catastrophic expenditure*.” “*Disaster relief*” was modified to “*disaster relief above what is required for license*.” “*Medicare shortfall*” and “*Medicaid shortfall*” were added and “*emergency preparedness or disaster relief*” as reworded to “*emergency preparedness above what is required for license*”. Lastly, “*environmental protection*” was changed to “*environmental protection that directly affect the health of population*, *above what is required for license*.” As presented in Panel B in Table 4, all suggested activities were supported by both Chinese and the U.S. experts with over 70 percent support rates, except *“high quality medical services*,” “*bad debt*,” “*adhering/complying with government mandates*,” and “*Tax payments or payments in lieu of taxes”* and “*Medicare shortfall”*.

**Table 4:** Comparisons Delphi results between China and USA.

Panel A: 2^nd^ Delphi results in China			Panel B: 2^nd^ Delphi results in the U.S.		
China Rank	HCB activity/ program	Score	USA Rank	HCB activity/ program	Score
**1**	Emergency preparedness or disaster relief	4.85	1	Charity care	4.71
**1**	Adhering/complying with government mandates	4.85	2	Emergency preparedness above what is required for license	4.36
**3**	Health promotion and health education	4.75	3	Disaster relief above what is required	4.29
**4**	Environmental protection	4.70	3	Social benefit activities	4.29
**4**	Education and research	4.70	5	Health promotion and health education	4.21
**6**	Charity care	4.65	6	Education and research	4.07
**6**	High quality medical services	4.65	7	Environmental protection that directly affect the heath of population, above what is required for license	4.00
**8**	Medical services with positive externality	4.45	7	Provision of low profit services	4.00
**9**	Provision of low profit services	4.35	9	Sliding scale fees based on income level	3.93
**10**	Social benefit activities	4.35	10	Medicaid shortfall	3.86
**11**	Bad debt	4.10	10	Medical services with positive externality	3.79
**12**	Discounted pricing	4.10	12	Bad debt to low-income group	3.57
**13**	Tax payments	3.65	13	Bad debt because of catastrophic expenditure	3.43
			14	Tax payments or payments in lieu of taxes	3.14
			15	High quality medical services	2.50
			16	Adhering/complying with government mandates	2.43

**Sources**: Study findings; China: 2010, USA: 2014

Providing high quality health service was regarded as a primary HCB activity and/or program for Chinese hospitals, even though it may not produce direct benefits for the community. In contrast, the U.S. experts did not support this activity and/or program as a HCB activity.

Social insurance shortfall, which was combined with bad debt was accepted as one of the HCB activities in China because the country has successfully achieved universal health insurance coverage −95 percent of the population are covered in 2011 [11]. However, in the U.S., Medicaid and Medicare shortfalls were separated into two HCB activities in the Delphi study. While Medicaid shortfall was accepted, Medicare shortfall was not considered as a HCB activity and/or program.

## Results and discussion

The comparative Delphi study between China and the U.S. demonstrates that despite differences in the healthcare context, there is high level of agreement between Chinese and the U.S. experts regarding what constitute as HCB. For example,4 of the 6 top-rated activities are similar between Chinese and the U.S. experts, including “*charity care*,” *“emergency preparedness above what is required for license*,” “*health promotion and health education”* and *“education and research”*.

However, there are some significant disagreements between the Chinese and U.S. experts as well. Chinese experts rated public health activities higher than the U.S. experts—“*adhering/complying with government mandates*” had the second highest support rate from Chinese experts, however, it was the lowest rated activity/ program by U.S. experts. Chinese experts categorized it as one of the most important HCB activity/ program with a score of 4.85 because the majority of Chinese hospitals are government-owned public hospitals [11]. This activity is used by the Ministry of Health as one of the hospitals’ performance measurements [43, 44]. Hospitals are encouraged to comply with government mandates such as supporting village visits, improving community health, and investing resources in foreign aid. In 150 hospitals where the content analysis was conducted, hospitals are mainly responsible for providing health care services to rural communities, second- or third-tier hospitals, or remote areas like Xinjiang and Tibet. For example, People’s Hospital-3 in the city of Luoyang assisted a community health clinic in providing its health services to rural communities, and People Hospital -1 in the city of Nanyang sent a team of providers to Xinjiang to provide technical assistance [45].

One major difference between Chinese and U.S. expert opinions is related to hospitals participation in “*emergency preparedness or disaster relief*”; Chinese experts rated it as the most important HCB activity/ program while the U.S. experts rated it as the third important. This discrepancy could be attributed to hospitals’ ownership: The U.S. non-profit hospitals are not considered as an extension of the local health department and how much they participate in public health activities depends on stakeholders’ decisions and whether there is a public hospital available in the county or city. Chinese hospitals however, are mostly financed by the local health department [46]. In China, this activity includes assembling care, rescue, and backup teams and conducting workshops or drills to handle the emergency [45]. For example, hospitals sent emergency care teams to treat patients with the flu during the H1N1 influenza outbreak. Hospitals from other provinces also provided health care support to hospitals and clinics in Sichuan province after the earthquake in 2008. Similarly, with “*environmental protection*,” U.S. experts only categorized this activity as HCB if hospitals provide these services “*above what is required for a license*”.

“*Health promotion and health education*” is ranked as the third most important HCB activity and/or program by Chinese experts, but is only ranked as the fifth by U.S. experts. In China, this activity includes planning for health promotion days such as “Love Your Eyes Day,” “Diabetes Day,” and “Protect Your Feet Day,”, as well as providing charity care services such as screening, physical check-up, and education workshops for rural and communities. As an example, People’s Hospital in the city of Penglai organized community service events to provide physical check-ups and diabetes prevention workshops for the elderly. In addition, several hospitals host regular forums and meetings for patients and their families to communicate with and learn from each other about the recovery process [45].

U.S. experts rated “*charity care*,” “*emergency preparedness above what is required for license*,” “*disaster relief above what is required*,” “*social benefit activities”* and *“health promotion and health education”* as the top five HCB activities. *“Charity care” was rated* as the top HCB activity because of the lack of universal coverage in the U.S. compared to China—many Americans remain uninsured or under-insured despite the passage of the ACA and continued health care reform efforts. Healthcare is considered as a “right” in China but in the U.S., except for emergency care, it is treated as a “commodity.” The political discussion around universal health insurance coverage remains complex and challenging. The enactment of the ACA has not settled the debate over the role of government in the provision of health insurance and healthcare delivery in the U.S. Policymakers are still struggling with how provide affordable healthcare to all residents, especially its most vulnerable populations.

In addition, in the U.S., being a non-profit hospital is often more lucrative compared to for-profits. Non-profit hospitals, which are presumed to be tax-exempt charitable organizations that provide much-needed community healthcare services or free/discounted services for those who cannot afford to pay, instead, reinvest their profits in the hospital in the form of more staff, more services, new facilities and equipment, or big salaries or bonuses for executives [47, 48]. Even though under the ACA, hospitals are required to conduct a community needs assessment every three years and develop strategies for meeting identified needs, the implementation or the level of charity services provided to the community vary significantly among non-profit hospitals. As a result, coverage gap remains for those who cannot afford to pay for their hospital services.

### Limitation

There was a lower response rate among the U.S. experts, which may result in the risk of selection bias. In addition, while the scholars in the study are national thought leaders. the study included the opinions of hospital administrator and policymakers from only one province in China (Jiangsu) and one U.S. state (Maryland). Hence, the results may not be representative of expert approaches in China and the U.S. Finally, there is a time lapse between the Chinese and American Delphi rounds. Two rounds of Delphi study were administered in 2010 in China while the other two rounds were conducted 2014 in the U.S. Some HCB activities may have evolved in both countries because of policy or hospital system changes.

## Conclusions

Several previous studies addressed different aspects of health system in the US and China, such as electronic health record [49], nursing [50], pharmacy education programs [51] and healthcare reform [52], however, this is the first cross-country comparison study on HCB and it has important implications.

This cross-country comparative study shows that despite different health care contexts, there are important agreements between China and the U.S.—more than 68.75 percent of activities received an over 70 percent support rate. There are also crucial lessons learned:

- Chinese experts believe that health care is a “right” of which the government has the main responsibility to provide needed healthcare services.
- The Chinese government should propose more specific policies for HCB guidelines, based on extensive HCB-related rules in the U.S.
- The approach validated the crucial role of government in the U.S., as an important player in providing coverage to vulnerable and low-income groups (e.g. the ACA).
- There is a need for the U.S. government to issue specific policies on the amounts/ types of community benefits non-profit hospitals should provide to receive tax exemption status.
- The scope and specific items for HCB activities/programs should stay flexible as the concept evolves over time.
- Certain HCB activities such as emergency preparedness or disaster relief, environmental protection, education and research, and high quality medical services and social benefit activities, are well-known activities in Organization for Economic Co-operation and Development (OECD) countries but are still under consideration in most of the developing countries. More attention need to be focused on these activities as they contribute to the public good and have significant impact on low-income people and communities.

## Supporting information

S1 Table Delphi techniques round-1 and round-2 raw data; China and USA. Please see PLOSONE-Data-China-US-9-12-19.xls for more information.
(XLS)
